# The Characteristics of Prostate Mucinous Adenocarcinoma Visualized by ^68^Ga-PSMA, ^68^Ga-FAPI, and ^18^F-FDG PET/CT

**DOI:** 10.1097/RLU.0000000000006004

**Published:** 2025-06-05

**Authors:** Zhuxu Sun, Xi Jiang, Guangbo Fu, Suan Sun, Weijing Tao

**Affiliations:** Departments of * Nuclear Medicine; † Urology; ‡ Pathology, The Affiliated Huaian No. 1 People’s Hospital of Nanjing Medical University, Huai’an, Jiangsu, China

**Keywords:** prostate mucinous adenocarcinoma, PSMA, FAPI, FDG, PET/CT

## Abstract

Primary prostate mucinous adenocarcinoma is an exceedingly rare entity with distinct imaging characteristics. We report the case of a 62-year-old man with biopsy-proven prostate mucinous adenocarcinoma. The patient underwent MRI and multi-tracer PET/CT. PET/CT images demonstrated the prostate lesion with moderate uptake of ^68^Ga-PSMA and ^68^Ga-FAPI, yet significantly high ^18^F-FDG uptake. No abnormal radiotracers’ uptake was observed elsewhere in the body. Multi-tracer PET/CT findings suggest that ^18^F-FDG may be a more suitable radiotracer for the systemic assessment of primary prostate mucinous adenocarcinoma.

**FIGURE 1:**
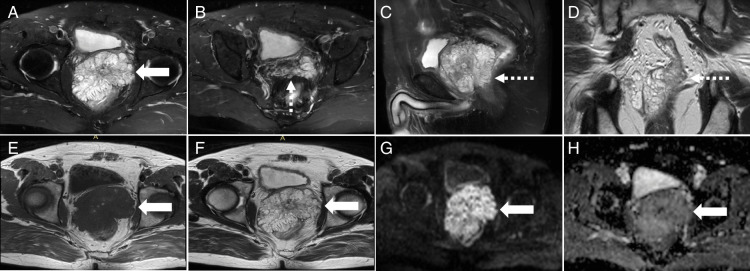
A 62-year-old man presented with progressive dysuria for 3 years without fever, hematuria, or abdominal pain. MRI revealed a multi-cystic mass in the prostatic region, measuring 9.2 × 6.3 × 8.1 cm (A, white arrow). Axial (B) and sagittal (C) fat-suppressed T2-weighted imaging (T2WI) and coronal T2WI (D) demonstrated a multi-septate lesion invading the seminal vesicles and anterior rectal wall (dashed arrows). The cystic fluid (white arrows) showed mixed hypo- and hyper-intensity on axial T1WI (E) and T2WI (F). Diffusion-weighted imaging (DWI) revealed hyper-intensity (G), while the apparent diffusion coefficient (ADC) map showed hypo-intensity (H), indicating restricted diffusion within the cystic fluid. These imaging features strongly suggested prostate mucinous adenocarcinoma.

**FIGURE 2:**
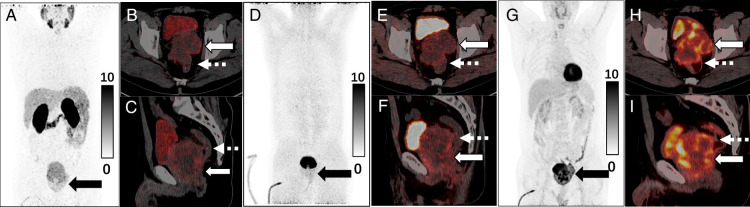
Multi-tracer PET/CT imaging was performed for preoperative systemic evaluation (Ethics No.: YX-2021-113-01). PET/CT maximum intensity projection (MIP, A) and axial (B)/ sagittal (C) images showed moderate ^68^Ga-PSMA uptake of the prostate lesion (SUV_max_: 7.17). Recognizing the “FAPI-high/FDG-low” imaging signature of mucinous adenocarcinomas, ^68^Ga-FAPI and ^18^F-FDG PET/CT were conducted. The lesion demonstrated moderate ^68^Ga-FAPI (D, MIP; E and F, PET/CT) uptake (SUV_max_: 12.75) and apparently higher ^18^F-FDG (G, MIP; H and I, PET/CT) uptake (SUV_max_: 25.46). Images revealed anterior rectal wall infiltration (dashed arrows) and confirmed the prostate lesion as the primary malignancy due to no extra-prostatic abnormal uptake. The marked FDG avidity, compared with PSMA and FAPI uptake, suggests a unique pathologic subtype of prostate cancer.

**FIGURE 3:**
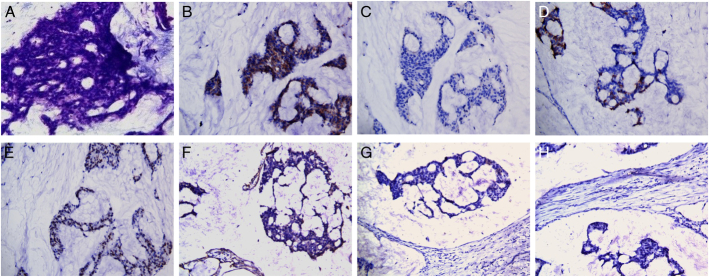
The patient underwent radical prostatectomy and partial rectosigmoid resection. Histologic examination with hematoxylin and eosin staining (A, ×200) revealed abundant extracellular mucin and scattered adenocarcinoma cells with a Gleason score of 7 (4+3). Immunohistochemistry showed the expression of mild P504S (B, ×200), negative PSA (C, ×200), moderate MUC2 (D, ×200), and moderate NKX3.1 expression (E, ×200). Analysis of tumor invasion and metastasis markers revealed the expression of partially positive FAP (F, ×200), weak MUC6 (G, ×200), and negative mutant p53 (H, ×200). Limited FAP expression correlated with mild ^68^Ga-FAPI uptake. Unlike mucinous adenocarcinomas in other organs with strong FAP overexpression and poor prognosis,^1,2^ this case showed weak FAP positivity, suggesting a potentially better prognosis consistent with existing literature.^1,3,4^ In summary, prostate mucinous adenocarcinoma, constituting 4% of all prostate adenocarcinomas, has abundant extracellular mucin and distinct immunohistochemistry profiles.^3,5,6^ For preoperative evaluation of this subtype, ^18^F-FDG outperforms ^68^Ga-FAPI/^68^Ga-PSMA due to higher uptake. This case underscores multi-tracer PET/CT’s diagnostic value for rare cancer subtypes and emphasizes the unique imaging and histopathologic features of prostate mucinous adenocarcinoma.^7,8^
